# Exploration of Vibrotactile Biofeedback Strategies to Induce Stance Time Asymmetries

**DOI:** 10.33137/cpoj.v5i1.36744

**Published:** 2021-10-29

**Authors:** R Escamilla-Nunez, H Sivasambu, J Andrysek

**Affiliations:** 1 Institute of Biomedical Engineering, University of Toronto, Toronto, Canada.; 2 Bloorview Research Institute, Holland Bloorview Kids Rehabilitation Hospital, Toronto, Canada.

**Keywords:** Gait, Human Movement, Biofeedback, Learning Effect, Motor Control, Rehabilitation, Short-term Retention, Symmetry Ratio, Vibrotactile Feedback, Wearable Systems

## Abstract

**BACKGROUND:**

Gait symmetry is the degree of equality of biomechanical parameters between limbs within a gait cycle. Human gait is highly symmetrical; however, in the presence of pathology, gait often lacks symmetry. Biofeedback (BFB) systems have demonstrated the potential to reduce gait asymmetry, improve gait function, and benefit overall long-term musculoskeletal health.

**OBJECTIVE(S):**

The aim of this study was to develop a BFB system and evaluate three unique BFB strategies, including bidirectional control – constant vibration (BC), bidirectional control – variable vibration (BV), and unidirectional control – variable vibration (UV) relevant to gait symmetry. The assessed feedback strategies were a combination of vibration frequency/amplitude levels, vibration thresholds, and vibrotactile stimuli from one and two vibrating motors (tactors). Learning effect and short-term retention were also assessed.

**METHODOLOGY:**

Testing was performed using a custom BFB system that induces stance time asymmetries to modulate temporal gait symmetry. The BFB system continuously monitors specific gait events (heel-strike and toe-off) and calculates the symmetry ratio, based on the stance time of both limbs to provide real-time biomechanical information via the vibrating motors. Overall walking performance of ten (n=10) able-bodied individuals (age 24.8 ± 4.4 years) was assessed via metrics of symmetry ratio, symmetry ratio error, walking speed, and motor's vibration percentages.

**FINDINGS:**

All participants utilized BFB somatosensory information to modulate their symmetry ratio. UV feedback produced a greater change in symmetry ratio, and it came closer to the targeted symmetry ratio. Learning or short-term retention effects were minimal. Walking speeds were reduced with feedback compared to no feedback; however, UV walking speeds were significantly faster compared to BV and BC.

**CONCLUSION:**

The outcomes of this study provide new insights into the development and implementation of feedback strategies for gait retraining BFB systems that may ultimately benefit individuals with pathological gait. Future work should assess longer-term use and long-term learning and retention effects of BFB systems in the populations of interest.

## INTRODUCTION

Human gait is a complex physical activity involving the primary motor and somatosensory cortices, as well as the spinal cord (i.e., central pattern generator for locomotion), and the musculoskeletal system.^1–3^ The interaction between the central and peripheral nervous systems, reflexes, muscles, and joints allows individuals to ambulate in a stable, synchronized, and symmetrical manner.^4^ Gait symmetry is the degree of equality of biomechanical parameters between limbs within a gait cycle.^4^ Able-bodied gait is typically characterized by a high degree of symmetry. However, neurological disorders or physical impairments such as Parkinson's,^5^ cerebral palsy,^6^ stroke,^7^ incomplete spinal cord injury,^8^ and lower limb amputation,^9^ can lead to pathological gait, resulting in atypical and asymmetrical gait patterns.^2^ Gait asymmetry can affect diverse biomechanical and physiological parameters.^9^ For instance, individuals with lower limb amputation often have reduced stance time support on the affected limb compared to the intact limb,^9^ as well as reduced walking speed, cadence,^10^ poor balance, and increased energy expenditure.^11^ Accordingly, the restoration of gait symmetry is critical for improving mobility, balance, function and efficiency, and overall long-term musculoskeletal health. Therefore, achieving gait symmetry is an important goal of gait rehabilitation.

Gait rehabilitation typically entails motor learning and providing verbal cues related to the patient's gait deviations or abnormal movement patterns to encourage positive changes. Gait rehabilitation is commonly provided by a physiotherapist, and the feedback is usually limited to subjective assessment of movement patterns. Additionally, rehabilitation sessions are often limited in duration and frequency.^12^ Patient barriers (e.g., long travel times, accessibility, etc.) and limited resources of healthcare facilities also often restrict access to physiotherapy.^12^ Technology-driven approaches, such as therapy-focused videogames,^13^ virtual reality,^14^ and biofeedback (BFB),^15,16^ have the potential to address the aforementioned challenges and provide alternative and augmentative means of training and rehabilitation in clinical settings or home-based environments. Specifically, BFB is the process of measuring physiological/biomechanical parameters and providing the user with real-time information about their current physical status.^17^ Wearable BFB systems for gait training can improve gait patterns by providing real-time, continuous feedback that reinforces good walking habits and physiotherapy goals.^16,18^

One of the challenges of using BFB for rehabilitation is the establishment of effective feedback strategies and modalities (i.e., how biomechanical information is communicated to the BFB user).^19,20^ Compact and wearable auditory and visual BFB systems are available;^16^ however, haptic BFB systems may be more suitable for field and community-based applications, since stimuli perception is less prone to be affected by external conditions such as noise or visual distractions.^15^ However, effectiveness of haptic modalities is highly dependent on the user's ability to sense, interpret, and appropriately respond to the vibrotactile signals. Previous studies have demonstrated the effectiveness of vibrotactile-based BFB systems to alter gait and assessed the impact of varying properties such as vibration amplitude and frequency, location of tactors, interfaces, and pressures on somatosensory response.^21–24^ However, few studies have applied a systematic approach to explore which BFB strategies most effectively achieve the desired symmetry targets. For instance, Afzal et al. tested different feedback strategies based on vibration durations and intensities, finding that greater alterations in gait symmetry occur with proportional vibrotactile feedback.^25^ Lee et al. demonstrated that continuous vibration (i.e., progressive modulation of tactor's intensity) performed better than an ON/OFF vibration approach during dynamic weight-shifting balance training of elderly and individuals with Parkinson's disease.^19^

While substantial research has been conducted toward developing BFB strategies, to the best of the author's knowledge, no study has attempted to compare and evaluate multiple vibrotactile biofeedback strategies based on gait symmetry targets, speed, and short-term learning effects when modulating gait symmetry. Hence, the overarching goal of this study was to develop a wearable vibrotactile BFB system and evaluate the effect of three unique BFB strategies on temporal gait symmetry and speed. In addition, aspects of learning and short-term retention effects were assessed by evaluating pre- and post-feedback gait parameters.

## METHODOLOGY

### A. System Instrumentation

**Figure 1:**
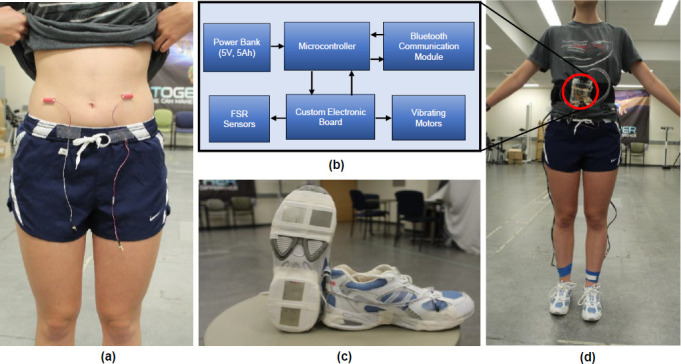
Main components of the wearable BFB prototype (vibrating motors, microcontroller, Bluetooth, power supply, and FSRs sensors). (a) Vibrating Unit (motors) located at the lower abdomen at the prolongation axis of the rectus femoris muscle. (b) Microcontroller-based Control Unit, including the custom electronic board, the communication (Bluetooth) module, and the power supply. (c) Sensors/Transducer Unit comprises four FSRs sensors located at the heel (x2) and toe (x2) of each shoe sole. (d) Set-up of the BFB system on a participant.

A BFB prototype system was developed that comprised of the following units (**Figure 1**). The Vibrating Unit (**Figure 1a**) included two vibrating motors (tactors) 9mm in diameter and 25mm in length (model 307-103, Precision Microdrive, United Kingdom). Each vibrating motor was supplied with 3.3V, corresponding to a nominal vibration frequency of 250Hz and vibration amplitude of 7.5g (i.e., g = 9.8m/s^2^, the gravity of Earth). Recent studies suggest that higher frequencies (>230Hz), targeting Ruffini cylinders and Pacinian corpuscles skin mechanoreceptors, increase user detection accuracy and reduce reaction times after vibrotactile stimulation.^22,23^ The vibrating motors were adhered to the lower abdomen (using surgical tape, TransporeTM, 3M Canada) at the prolongation axis of the rectus femoris muscle following previous studies.^26,27^ The Microcontroller-based Control Unit (**Figure 1b**) was comprised of the Arduino Uno Rev3 (Sparkfun Electronics; Boulder, Colorado, USA) and a custom electronic board with N-type MOSFETs, diodes, and resistors, which was designed to ensure the correct operation and power supply of the vibrating motors and the Sensors/Transducer Unit. The Sensors/Transducer Unit (**Figure 1c**) included four-square force sensitive resistors (FSRs) (model 406, Interlink Electronics, USA) to detect foot contact (heel-strike and toe-off). The FSR's force sensitivity ranged from 0.2N to 20N. Eight FSR sensors (four per foot) were adhered to the shoe sole, underneath the heel (x2) and the 1st and 5th metatarsal heads for the toe (x2). The Power Supply consists of a 5V at 5Ah Lithium-ion battery (PowerCore 5000 by Anker Innovations, Shenzhen, China) powered the entire system. The Communication module included a Bluetooth serial communication device (HC-05 Bluetooth module by Smart Prototyping, Hong Kong) which provided wireless communication between the microcontroller and a host PC.

An open-source software, Tera Term (Tera Term Project, Japan) was utilized for real-time data acquisition and visualization on the host PC at a sampling rate of 100 Hz (10ms resolution). A set-up of the BFB system on a participant is shown in **Figure 1d**.

### B. Biofeedback System Operation

**Figure 2:**
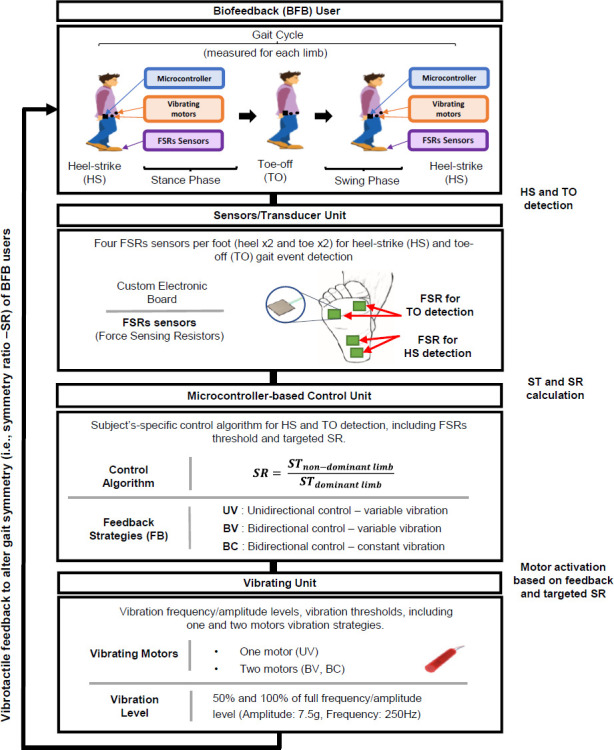
Control diagram of the BFB system to modulate symmetry ratio (SR). The closed-loop system includes system components, feedback strategies, and system operation.

The BFB system employs a closed-loop design to continuously monitor specific gait events, namely heel-strike (HS) and toe-off (TO) (**Figure 2**). Thus, the symmetry ratio (SR) was calculated to provide real-time biomechanical information via the vibrating motors to alter SR of BFB users. FSR thresholds for HS and TO onsets were determined by using a peak detection algorithm presented by Lopez-Meyer et al.^28^ The detection of HS and TO was used to compute the stance time (ST) of each leg. ST was defined as the amount of time that each leg remains in contact with the ground during each gait cycle.^29,30^ Subsequently, SR was used to quantify gait symmetry based on the Equation:^29,30^

$$SR=\frac{ST_{non-dominant\;limb}}{ST_{dominant\;limb}}$$

where, ST denotes the stance time of the non-dominant and dominant limbs, respectively. SR was measured for each limb and used to provide feedback during the stance phase of the subsequent step.^29,30^ For non-pathological gait, SR values typically range between 0.95 to 1.05.^25^ Vibrotactile feedback was delivered to users based on the selected feedback strategies (i.e., a combination of vibration levels, vibration thresholds, and control algorithms for one and two motors activation strategies) as detailed below.

### C. Biofeedback Strategies

Three novel strategies were applied that consisted of different vibration/amplitude levels, and number of stimuli (i.e. one or two motors). The three feedback strategies included **1)** Bidirectional control – constant vibration (BC), **2)** Bidirectional control – variable vibration (BV), and **3)** Unidirectional control – variable vibration (UV).

**Figure 3:**
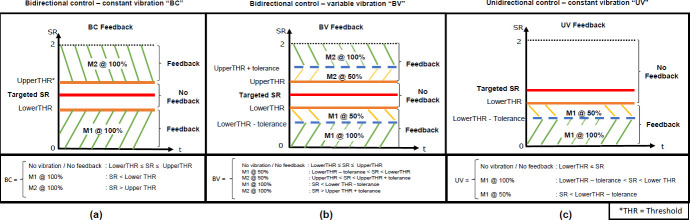
Vibrotactile feedback strategies. (a) BC: Bidirectional control – constant vibration; (b) BV: Bidirectional control – variable vibration; and (c) UV: Unidirectional control – variable vibration. BC utilizes two motors (M1 & M2), which produce vibrations at 100% of full vibration frequency/intensity. BV utilizes two motors, which produce vibrations at 50% and 100% of full vibration frequency/intensity. And, UV utilizes only one motor (M1), which produces vibrations at 50% and 100% of full vibration frequency/intensity. Motors “M1” and “M2” were placed at the left and right side of the lower abdomen at the prolongation axis of the rectus femoris muscle, respectively. Vibrating thresholds are denoted by LowerTHR and UpperTHR for lower and upper thresholds, respectively. The desired SR value is denoted by Targeted SR. A tolerance of 0.05 was selected based on typical SR values for individuals with non-pathological gait (i.e., 0.95 ≤ SR ≤ 1.05, where SR=1.0 denotes perfect gait symmetry).

Bidirectional control (BC and BV) provides feedback when the targeted SR value is either exceeded or not achieved (**Figure 3a&b**). Unidirectional control (UV) feedback only provides feedback if the targeted SR value is not achieved (**Figure 3c**). The unidirectional control uses a single vibrating motor, and bidirectional uses two motors (**Figure 3**). Vibrating motors are activated at two different vibration levels. A greater deviation from the targeted SR produces a vibration at 100% power, and a smaller error produces vibrations at 50% power. It should be noted that the magnitude of the SR error (i.e., the difference between the targeted SR and the currently measured SR), the pre-set vibration thresholds, and the targeted SR determine the activation and vibration level of the motors according to the applied feedback strategy (**Figure 3**). A tolerance equal to 0.05 was selected based on typical SR values for individuals with non-pathological gait (i.e., 0.95 ≤ SR ≤ 1.05, where SR=1.0 denotes perfect gait symmetry)^25^ (**Figure 3**). The UV strategy is designed to encourage the BFB user to move toward and exceed the SR target, at which point the vibrotactile feedback stops (**Figure 3c**). Strategies BC and BV require the BFB user to maintain SR within specified thresholds (**Figure 3a&b**).

Previous studies suggest that permanent gait changes must be achieved gradually,^30^ finding that absolute differences larger than 5% can be considered as potential improvement or deterioration of gait symmetry.^31^ For this reason, all feedback strategies (BC, BV, UV) targeted SRs that were 10% greater than an initial SR baseline (e.g., mean initial SR baseline = 1.0; targeted SR = 1.10), as proposed by.^25^ SR converging towards 1, indicates an improvement in gait symmetry, whereas SR diverging from 1, indicates a deterioration.^31^ Since this study involved participants with non-atypical gait symmetry (i.e., SR = 1 ± 0.05), the testing paradigm consisted of deviating SR from SR = 1 to alter gait symmetry. For all the feedback strategies, vibrations (if provided) start at HS and end at TO of the same limb within a gait cycle. Vibrations are only provided if participants are walking with a SR value outside of the pre-set vibrating thresholds (**Figure 3**).

### D. Participants

The study involved a convenience sample of ten (n=10) healthy subjects (five males), age 24.8 ± 4.4yrs; height 1.7 ± 0.1m; weight 68.7 ± 14.4kg. Participants were 18 years or older, were all English speaking, and having no physical or gait-related impairments, ambulation difficulties or neuro-motor disorders. The study was approved by the Research Ethics Board (REB #16-675) at Holland Bloorview Hospital, Canada. Informed written consent was obtained from each participant before commencing.

### E. Experimental Protocol

**Figure 4:**
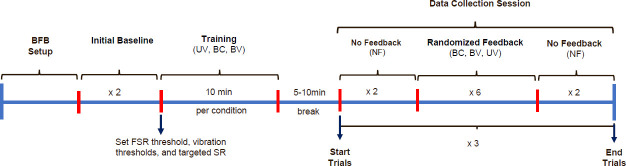
Experimental protocol for the data collection session. Baseline trials consist of wearing the BFB system, but no feedback (NF) is provided. Feedback strategies (BC, BV, UV) were randomized for data collection. BC: Bidirectional control – constant vibration, BV: Bidirectional control – variable vibration, and UV: Unidirectional control – variable vibration.

Data were collected in a single session. Participants were instrumented with the BFB system (**Figure 1**). Training was provided before collecting data (**Figure 4**). Training consisted of a brief explanation about the BFB operation and the opportunity to walk using each feedback strategy (BC, BV, UV). During training, participants were coached about how to interpret the vibrotactile feedback. To determine limb dominance, participants were asked about what foot they use to kick a ball. All participants were right footed (i.e., right limb was the dominant limb). For the UV strategy, the vibrating motor (M1) was placed on the non-dominant (left) side at the lower abdomen level. For the BV and BC strategies, motor M1 was placed on the non-dominant (left) side and the second motor (M2) on the dominant (right) side at the lower abdomen. Since the goal was to achieve a 10% change in SR (i.e., increase ST on the non-dominant “left” limb), verbal instruction and cues were provided as follow: for UV strategy (motor M1 placed on the non-dominant side), “*if the motor on your left side vibrates, you need to spend more time in contact with the ground on that (left) side*”. Hence, for BV and BC strategies, a verbal instruction/cue for motor M2 was provided in addition to the one provided for M1, “*if the motor on your right side vibrates, you are spending too much time on your left side, so you just need to spend a little bit less time in contact with the ground on your left side*”. In terms of the vibration levels, cues consisted of “*while walking, you will experience two different vibration intensities, the weaker vibration means you are closer to the target, and stronger means you are farther from the target. The goal is to receive no vibration*”. For the data collection, each participant performed 30 walking trials in total (i.e., 4 No Feedback trials plus 6 Feedback trials for each of the three strategies). Each trial consisted of walking 20 meters in a straight line at a self-selected speed. Feedback strategies (BC, BV, UV) were randomized using simple and balanced randomization through a random number generator. No feedback “NF” condition was performed before (x2 trials) and after (x2 trials) each feedback strategy. Both NF conditions (before and after feedback) were used as a baseline to compare BFB effects within strategies. Average gait speed was calculated for each trial based on the walking distance and time recorded with a stopwatch. The magnitude of the SR error was calculated for each gait cycle as the difference between the targeted SR and the measured SR. The percentage of vibration was calculated based on the activation status of each motor, which indicates the number of times that the motors were activated (ON = 1) or deactivated (OFF = 0) for each trial (i.e., time (ON/(ON + OFF))*100).

### F. Data Analysis

Data recorded and captured using TeraTerm software (Tera Term Project, Japan) were exported to Excel (Microsoft Corp; Redmond, Washington) and processed in MATLAB 2019b (R2019b, Mathworks, MA, USA) to extract parameter values (i.e., SR, SR error, average gait speed, and percentage of vibration) for each trial, condition, and participant. Statistical analysis was performed using JMP Pro 2019 software (Statistical Discovery, SAS, USA). A Shapiro-Wilk W Test with an alpha level of 0.05 was used to confirm the assumption of normal distribution of the data. A multivariate analysis of variance (MANOVA) was performed between and within participants, trials, and conditions (NF, BC, BV, UV) across all retrieved parameters. Statistical significance was determined using a critical alpha level of 0.05 for all primary analyses. If statistically significant differences were found, a Fit Model – Mixed Model analysis with a post-hoc Tukey HSD all pairwise comparisons analysis was performed to identify which particular differences between pairs of means were significant. In addition, a paired t-test was performed to compare differences on the level of precision with which the targeted SR was achieved between conditions (NF, BC, BV, UV). For this test, the standard deviation of the SR values was used. Finally, a paired t-test was used to compare SR values between NF conditions (i.e., NF before and after vibrotactile feedback) to identify statistically significant short-term retention effects. A Bonferroni correction with an adjusted critical alpha level of 0.008 (p = 0.05/6) was applied to reduce type I errors for multiple pairwise comparisons.

## RESULTS

### A. Biofeedback Strategies Effectiveness

**Figure 5:**
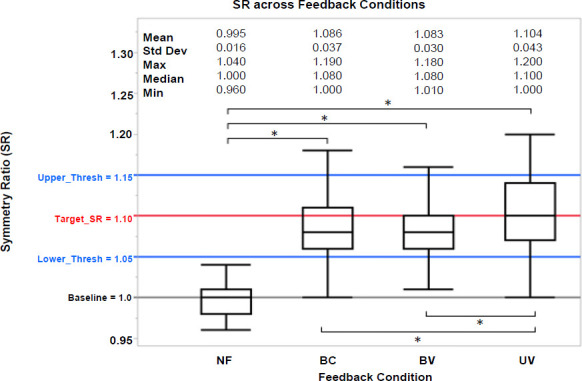
SR vs FB. Box plot of SR values for all feedback (BC, BV, and UV) and no feedback (NF) conditions. BC: Bidirectional control – constant vibration; BV: Bidirectional control – variable vibration; UV: Unidirectional control – variable vibration. Statistically significant differences between conditions are denoted with an ‘*’. Mean, standard deviations (Std Dev), maximum, median, and minimum values of SR are presented. Upper and Lower Thresholds labeled as Upper_Thresh and Lower_Thresh, respectively.

**Figure 6:**
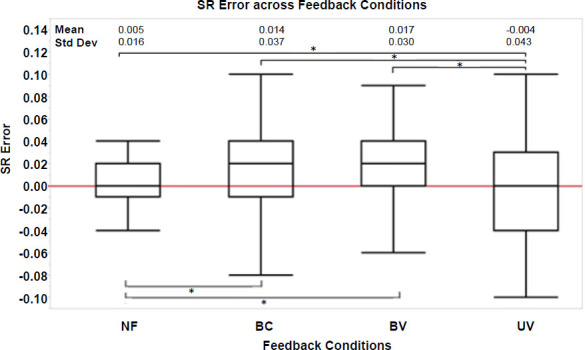
SR Error. Mean SR error values across feedback (BC, BV, UV) and no feedback (NF) conditions. BC: Bidirectional control – constant vibration; BV: Bidirectional control – variable vibration; UV: Unidirectional control – variable vibration. Statistically significant differences were found between NF and feedback conditions (BC, BV, and UV), including between feedback strategies UV-BC and UV-BV, but not between BC – BV strategies. Statistically significant differences are denoted with an ‘*’. Mean and standard deviations (Std Dev) are listed above.

Changes on SR values were found statistically significant when comparing between the feedback strategies (BC, BV, UV) and the no feedback (NF) condition (p < .001). Significant differences were also found within feedback strategies. UV produced a larger change in SR than BV (p < .001) and BC (p < .001) (**Figure 5**). However, changes in SR values between strategies BV and BC were not significantly different (p = 0.708). In terms of the SR error, all BFB strategies resulted in larger SR errors that were statistically different from NF (NF–BC: p < .001; NF–BV: p < .001; and NF–UV: p < .001) (**Figure 6**). There were also statistically significant differences among the SR errors for BC–UV (p < .001) and BV–UV (p < .001) strategies, but not among BC–BV (p = 0.512). Further, when comparing the standard deviation of SR, NF achieved a higher level of precision (smaller variability) compared than all the feedback strategies (p < .001; BC, BV, UV). However, no significant differences in the level of precision (i.e., standard deviation of SR) were found between feedback strategies (BC-UV: p = 0.057; BC–BV: p = 0.065; and BV–UV: p = 0.752).

### B. Short-term Retention and Learning Effects

**Figure 7:**
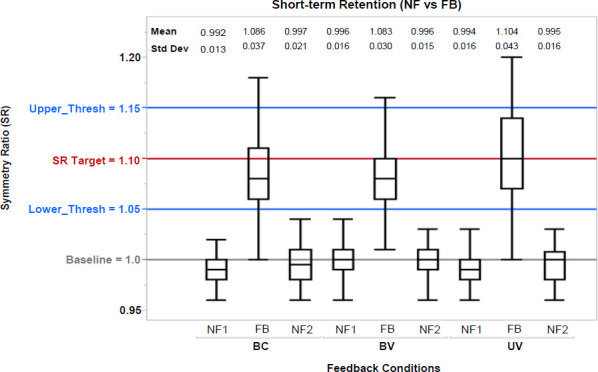
Short-term Retention. Box plot of SR values during No Feedback (NF) before (i.e., NF1) and after (i.e., NF2) providing vibrotactile feedback (BC, BV, UV). BC: Bidirectional control – constant vibration; BV: Bidirectional control – variable vibration; UV: Unidirectional control – variable vibration. Statistically significant differences were found only between NF and feedback conditions. Mean and standard deviations (Std Dev) are also included above. Upper and Lower Thresholds labeled as Upper_Thresh and Lower_Thresh, respectively.

In terms of short-term retention, no significant effects (p = 0.156) were evident from **Figure 7**, and based on the paired t-test statistical analysis of NF before (i.e., NF1) and after (i.e., NF2) vibrotactile feedback (BC, BV, UV).

**Figure 8:**
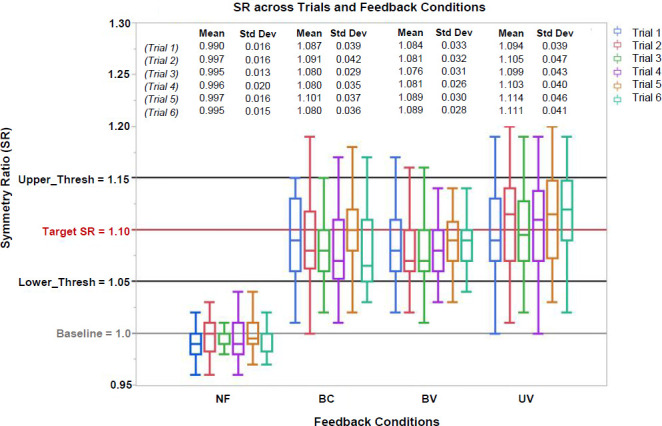
Learning Effects. Box plot of SR values for mean SR values of all participants across trials during No Feedback (NF) and Feedback conditions (BC, BV, UV). BC: Bidirectional control – constant vibration; BV: Bidirectional control – variable vibration; UV: Unidirectional control – variable vibration. No statistically significant differences were found among conditions. Mean and standard deviations (Std Dev) are also included above for each trial. Upper and Lower Thresholds labeled as Upper_Thresh and Lower_Thresh, respectively.

Learning effects for trial*feedback interactions within subjects were not statistically significant (**Figure 8**). However, for BV and UV strategies, participants neared the targeted SR from the first trial, with a slight but non-significant trend towards improvement in subsequent trials (**Figure 8**).

### C. Secondary Outcomes

All feedback conditions were associated with significantly slower walking speeds compared to the NF condition which was 1.398 m/s ± 0.022 (p < .001). When comparing walking speed between feedback strategies (BC, BV, UV), participants walked significantly faster with UV feedback (1.206 ± 0.029 m/s, p < .001) than BV (1.067 ± 0.023 m/s, p < .001) and BC (1.053 ± 0.020 m/s, p < .001). No significant differences in walking speeds were found between BV and BC (p = 0.900).

The percentage of vibration was significantly smaller under UV feedback (21%) compared to BV (27%), and BC (52%). The post-hoc Tukey HSD analysis showed a significant vibration percentage difference between conditions BC and UV (p < .001) and, BC and BV (p < .001). There was no significant difference in the vibration percentage between UV and BV (p = 0.959).

## DISCUSSION

The purpose of this study was to investigate the effect of BFB on SR and walking speed via three novel vibrotactile feedback strategies (BC, BV, UV), which are different combinations of vibration thresholds, vibration levels (frequencies/amplitudes), and control strategies based on the activation of one and two motors. These feedback strategies were utilized to provide somatosensory information to BFB users to modulate gait symmetry during walking. In addition, learning effects and short-term retention were investigated.

During walking trials, all participants were able to utilize BFB somatosensory information to alter their gait performance towards the targeted symmetry ratios (SR). Accordingly, results showed that BC, BV, and UV feedback strategies can all potentially modulate SR of BFB users.

The results also suggest that a unidirectional strategy (UV) can produce a greater change in SR, to bring it closer to the target value (lower SE error). Hence, UV more accurately achieved the target SR as compared to both BC and BV. While the precision (variability) was not significantly different among feedback strategies, based on **Figure 5** and **Figure 6** the bidirectional strategies (BC and BV) trended toward more precise changes in SR (i.e., error bars are larger for UV compared to BC and BV). This may be due to the target being exceeded more frequently (i.e., SR higher than set target). In contrast, the bidirectional strategies (BC and BV) having both an upper and lower limit around the target values, produced less variability. It must be noted that among all of the conditions, participants achieved the lowest SR variability in the NF condition. One approach to achieve both accuracy and precision would be to use a bidirectional strategy with an adaptive targeted SR control, by which the target SR is gradually increased as the user changes their SR. This technique may reduce variability by guiding users to perform smaller step-to-step increments.

Few differences were evident between the variable and constant bidirectional strategies (i.e., BV and BC, respectively), suggesting limited effectiveness in using vibration levels for thresholding. In this study the two distinct levels of vibration (both frequency and amplitude) were applied based on the magnitude of the error and pre-set vibration thresholds. Previous studies have resorted to altering duration of vibrations;^27,30,32,33^ and, only few of them have used variable amplitudes.^19,25^ In terms of the effectiveness of the vibration pattern (i.e., continuous versus corrective versus ON/OFF or discrete feedback), the previous research findings are mixed. Some studies suggest that continuous feedback (i.e., progressively incrementing or decrementing motor's intensity) produce greater gait improvements over discrete feedback (i.e., motors ON/OFF activation);^19^ and vice versa.^27,34^ Whereas, others suggest that corrective feedback (e.g., vibration only if targeted value is not reached) can elicit greater effects compared to continuous feedback (e.g., vibration until targeted value is reached).^32^ The present study combines discrete signals (ON/OFF) with corrective feedback, adding multiple vibration levels and thresholds, which provides the BFB system with unique feedback strategies to modulate gait symmetry of BFB users.

In this study we found the BFB learning effects and short-term retention to be minimal. According to the literature, learning a new skill or eliciting a locomotor adaptation is a complex process that involves motor adaptation, skill acquisition, and decision-making.^35,36^ The process of motor learning occurs gradually and improves over time. The learning process, at early stages, demands high cognitive effort, high consciousness of the task performed, and greater amounts of energy. However, at later stages, the movements seem to occur more unconsciously, automatically, and with less effort.^36^ It is plausible that extended use of the BFB system may result in relearning, and retention whereby the modified gait patterns would be preserved once the BFB system is no longer active. For instance, a recent study showed that 1 out of 3 above-knee amputee participants were able to retain improvements in gait symmetry (+14.9% improvement compared to baseline) after three training sessions of using vibrotactile feedback, suggesting an effective motor learning at least in the short-term.^37^

The slower than normal walking speeds associated with the provision of BFB, indicate potential limitations in terms of BFB effectiveness. However, it is foreseeable, that over the longer term as users utilize BFB less consciously, walking speeds may naturally recover towards normal values. Moreover, when feedback was provided, UV feedback achieved a significantly faster walking speed compared to BV and BC. UV also resulted in less vibrotactile feedback (vibration percentage) compared to BV and BC. Together, these results may indicate that as the complexity of the feedback strategy and information provided to BFB user increases, thus taxing of the executive function (i.e., cognitive processes), slower execution of function (e.g., slower motor response to stimulation) results. It might be beneficial for new BFB users to start the gait retraining with UV feedback. Once UV is learned, treatment can move progressively to BV or BC strategies.

Future iterations of the BFB system should incorporate visual or auditory feedback modalities to assess the effectiveness of multimodal feedback paradigms. In addition, future studies should have in mind that individuals with poor somatosensory function might have decreased sensory perception to stimulus detection, which might affect the performance of haptic BFB systems. However, the stochastic resonance phenomenon, which consists of delivering sub-threshold noise to the somatosensory system might be a promising alternative for enhancing sensitivity to sensory inputs,^38^ and for improving reaction times.^39^ Accordingly, traditional haptic BFB systems can be combined with a noise-generating device/module to enhance BFB sensory perception to improve gait asymmetries. Also, the effects of haptic BFB systems on the tonic vibration reflexes (i.e., reflex muscular contraction) and the excitatory and inhibitory responses of the muscle spindle, which play a role enhancing muscle activation should be further investigated, since acute indirect vibrations acting on muscles can potentially enhance force, power, flexibility, balance, and proprioception, which might suggest neural enhancement.^40^

This study has several limitations. The limited sample size of healthy subjects with non-asymmetrical gait represented a main limitation for generalizable conclusions. Thus, the performance of the BFB system in populations with pathological gait remains to be studied. Additionally, pressure sensors had a tendency to degrade over time resulting in inconsistent measurements. In such instances where measurements became unreliable, sensors were replaced, and additional data collected within the session. However, the development of a clinically relevant system will require more robust sensing instrumentation. To address the measurement issues with the FSRs, and also improve wearability, inertial measurement units should be considered. A final potential limitation relates to the sound generated by the vibrating motors. It is possible that it may have contributed to the feedback received by the participants. Thus, using headphones to cancel external sources of noise might be a point of consideration for future studies involving haptic feedback. As part of future work, secondary/indirect changes in gait patterns (either improvements or detriments) due to BFB should be investigated. Finally, longer-term use of BFB is needed to assess learning and retention effects.

## CONCLUSION

In this paper, the development of a wearable vibrotactile BFB system was presented along with the evaluation of three novel feedback strategies to modulate temporal gait symmetry by inducing stance time asymmetries. Clinical testing of the BFB prototype showed its ability to alter SR during walking; however, no learning effects or short-term retention effects were found.

## DECLARATION OF CONFLICTING INTERESTS

The authors declare no conflict of interest. The funders had no role in the design of the study; in the collection, analyses, or interpretation of data; in the writing of the manuscript, or in the decision to publish the results.

## AUTHOR CONTRIBUTION

- **Rafael Escamilla-Nunez:** Conceptualization, methodology, validation, formal analysis, investigation, writing—original draft preparation, writing/review and editing, visualization, funding acquisition.
- **Harry Sivasambu:** Validation, formal analysis, investigation, writing/review and editing, visualization.
- **Jan Andrysek:** Supervision Conceptualization, methodology, investigation, writing/review and editing.

## SOURCES OF SUPPORT

This research was funded by Natural Sciences and Engineering Research Council of Canada (NSERC) Discovery RGPIN 2018-05046, NSERC CRD CRDPJ 491125 – 15, and Mexico's National Council for Science and Technology (CONACyT).

## ETHICAL APPROVAL

The study was approved by the Research Ethics Board (REB #16-675) at Holland Bloorview Hospital, Canada. Informed written consent was obtained from each participant before commencing.
